# Impact of Livelihood Diversification on Rural Households’ Food and Nutrition Security: Evidence from West Shoa Zone of Oromia Regional State, Ethiopia

**DOI:** 10.1016/j.cdnut.2024.104521

**Published:** 2024-12-10

**Authors:** Firafis Haile, Jema Haji Mohamed, Chanaylew Seyoum Aweke, Terefe Tolessa Muleta

**Affiliations:** 1 School of Rural Development and Agricultural Innovation, Haramaya University, Dire Dawa, Ethiopia; 2 School of Agricultural Economics and Agribusiness, Haramaya University, Dire Dawa, Ethiopia; 3 School of Natural Resource, Ambo University, Ambo, Ethiopia

**Keywords:** food security, multinomial endogenous switching regression, nonfarm diversification, off-farm diversification, nutrition security

## Abstract

**Background:**

Food and nutrition insecurities continue to be significant issues for communities in developed and developing countries, even when there are plentiful harvests. In Ethiopia, climate change and other human-induced challenges are key factors contributing to this insecurity. Research and development experts suggest that implementing sustainable livelihood diversification strategies could be a viable solution.

**Objectives:**

The objective of the study was to analyze the determinants of choice of livelihood diversification strategies and its impact on food and nutrition security among smallholder farmers in the West Shoa zone, Oromia region, Ethiopia.

**Methods:**

The research employed quantitative approaches for data gathering and analysis. A multistage sampling method was utilized to choose the study locations. A total of 385 smallholder farming households (215 diversifiers and 170 nondiversifiers) were randomly chosen as participants from the 2 districts and 7 rural villages in the area. Descriptive statistics (frequencies, percentages, and cross-tabs) and econometric models [multinomial logit model and multinomial endogenous switching regression (MESR) model] were employed to analyze the quantitative data.

**Results:**

More than half (56%) of the farming households were able to diversify their livelihoods, whereas the remaining 44% were unable to do so, indicating a lack of means to engage in any form of livelihood diversification activity beyond agriculture. The results of the multinomial logit regression model revealed that various factors such as gender, age, family size, education level, farm experience, social norms, land ownership, livestock possession, access to credit, access to extension services, working capital, government policies, climate variability, livelihood training, and proximity to markets significantly influenced smallholder farming households’ choices and adoption of diversification strategies. The MESR model demonstrated that engaging in farming and off-farming activities could increase food and nutrition security for farm households by 74.6% and 33.3%, respectively. Similarly, participating in farming and nonfarming activities was associated with a 71.3% improvement in food security and a 42.3% enhancement in nutrition security. However, combining farming with both nonfarm and off-farm activities did not have a significant impact on food security, but it did lead to a 15.2% increase in nutrition security.

**Conclusions:**

Involving smallholder farmers in livelihood diversification reduces poverty, food insecurity, and unemployment. This study shows that diversifying livelihoods positively impacts food and nutrition security by enabling farmers to produce more for consumption and income generation.

## Introduction

Food security is defined as the condition in which “all individuals have access to enough safe and nutritious food to meet their dietary needs for an active and healthy life” [1]. Nutrition security status is the body’s ability to absorb and use nutrients, influenced by environmental factors such as food consumption and food security [1]. Approximately 800 million people lack access to sufficient food, over 2 billion suffer from essential micronutrient deficiencies, and ∼60% of individuals in low-income countries face food insecurity. The consequences of food and nutrition insecurity include negative impacts on physical, social, emotional, and cognitive development across the lifespan, as well as significant disruptions to both society and the environment, posing serious threats to planetary health [2]. Nowadays, the concept of food and nutrition security is a critical global development concern. It affects everyone, but developing countries have the most to lose [3,4].

Functional Food Science in Europe characterizes functional food as foods that not only provide nutrition but also offer ≥1 positive impact on the human body, aiming to reduce risk of disease [5]. A nutritious diet, whether natural or fortified, can improve the performance and productivity of individuals across different age groups and specific demographics [[6], [7], [8]] Research conducted by Tripathi and Jain [9] has shown that incorporating plant-based components into functional diets can significantly enhance human health. Achieving food security requires a holistic approach, including social protection measures to ensure access to safe and nutritious food, especially for children, and transforming food systems for a more inclusive and sustainable global environment [10]. It is essential to allocate resources to both rural and urban areas and implement social protection measures that ensure disadvantaged individuals have adequate access to food and thus improving overall quality of life [10].

The United Nations Organization predicts that over 600 million individuals will be affected by hunger globally by 2030, highlighting significant obstacles to the complete eradication of hunger. Despite progress toward achieving “Zero hunger and malnutrition” as outlined in Sustainable Development Goal-2 of the 2030 Agenda, the world is falling behind, the number of food insecure and malnourished individuals continues to rise [[11], [12], [13]]. According to FAO [12], the number of people suffering from chronic hunger worldwide in 2022 was 735 million up from 7.9% in 2019 to ∼9.2% in 2022. With the expected population growth, urbanization, and changing consumption habits, food production will need to increase in the world by 70% to accommodate an additional 2.3 billion people by 2050 [12]. It is somewhat paradoxical that many individuals experiencing undernourishment, those who cannot afford nutritious diets, are actually involved in food production, such as subsistence farmers and farm laborers [14].

Climate change exacerbates disasters worldwide, disproportionately impacting women, infants, and children. It is essential to prioritize their food security and nutrition to mitigate these effects. The combination of food insecurity and famines, exacerbated by climate change, hinders access vital nutrition and healthcare, putting health and development at risk [15]. Failure to meet the nutritional needs of pregnant women can adversely affect their health and increase the likelihood of delivering low birth weight infants [16], leading to long-term negative health outcomes for their children. Extreme events such as hurricanes, tornadoes, floods, prolonged droughts, and wildfires significantly affect public health causing issues such as food shortages, malnutrition, water and air pollution, increased mortality rates, and heightened poverty because of the disruptions in livelihoods [17]. The majority of those displaced by climate change are in developing countries, with women and children facing particular vulnerability. Displaced women are at a higher risk of gender-based violence, including domestic abuse, forced marriage, and trafficking [18]. However, current United Nations and government agendas do not adequately address the disproportionate impact of climate change on the health, food security, and nutrition of women, infants, and children in low-income settings [16,18,19].

In Sub-Saharan Africa (SSA), an estimated 346 million people are undernourished; although the percentage of those facing food insecurity expected to decrease from 40.5% in 2020 to 24.4% in 2030, SSA is predicted to have the slowest improvement in food security [20]. The high levels of food and nutrition insecurity in Africa can be attributed to various factors, including low agricultural productivity, challenging climatic conditions, slow economic growth, inadequate governance, shrinking land sizes because of population pressure, and limited research and innovation [21]. In Ethiopia, the number of people facing food insecurity rose dramatically from 5.6 million in December 2016 to 37.7 million in December 2023 [12]. This increase is primarily because of factors such as drought, conflict in northern Ethiopia, insecurity, post-harvest loss, crop diseases, and adverse climate conditions [[22], [23], [24]].

Access to sufficient, safe, and nutritious food is a basic human right yet many people around the world still lack access to adequate food. Addressing food and nutrition security is crucial for achieving sustainable development goals and ensuring a healthy and prosperous future for all [25]. Despite its significance, there is still confusion and misunderstanding about the definition of food security, its relationship to nutrition security, and the necessary frameworks for research and practice in this field [26]. To achieve food and nutrition security, consistent access to healthy, nutritious, and sustainable diets is essential. These diets should prioritize unprocessed or minimally processed foods such as fresh fruits and vegetables, whole grains, and sustainable protein sources, whereas reducing the consumption of ultraprocessed foods and beverages. It is also crucial to prepare these foods in healthy ways and ensure adequate intake of water [25].

As in many other African countries, there is a pressing need to improve household food and nutrition security in Ethiopia [27]. In Ethiopia, food and nutrition insecurity is closely linked to a heavy reliance on unvaried livelihoods centered on low-input and low-output rain-fed agriculture [28]. Agriculture serves as the main source of food and income for numerous rural households in Ethiopia, making it a crucial element in efforts to reduce poverty and achieve food and nutrition security. Despite the presence of various agricultural policies, Ethiopia’s agricultural production is considered inadequate, with a focus mainly on on-farm agricultural development [29,30]. The agricultural sector faces various challenges such as climate change, reliance on traditional farming methods, shrinking land sizes because of population growth, limited resources, declining soil quality, lack of access to modern agricultural technologies, and financial constraints. These challenges hinder agricultural productivity, leading to food and nutrition insecurity as well as poverty [12]. Consequently, rural households are compelled to adopt diversification strategies to address the growing vulnerability associated with agricultural production [31,32].

Diversifying income sources and livelihood options is essential to supplement farm income and reduce risks associated with agriculture, particularly in countries such as Ethiopia where agriculture is vulnerable to climate shocks [33,34]. Farmers are increasingly exploring alternative livelihood options to enhance their well-being, solely depending on agriculture may not be sustainable [33,[35], [36], [37]]. Despite the emphasis on agriculture, productivity remains low in Ethiopia, promoting many farm households to buy cereals instead of growing them [37]. The country’s dependence on subsistence farming, which is prone to climate shocks, highlights the importance of diversifying livelihoods to prevent asset depletion and alleviate poverty levels [[38], [39], [40],^41^]. Thus, without off/nonfarm activity diversification, increasing agricultural productivity and redressing the issues of access to key agricultural technologies could not successfully alleviate poverty and food insecurity. Hence, livelihood expansion, which aims to reduce the threat allied with agricultural production and supplement farm income, is highly needed [37].

Scholars have intensively investigated the impact of livelihood diversification on various outcomes. Ahmed and Melesse [41], Natnael [42], and Shako et al. [43] focused solely on food security without considering its relationship with livelihood diversification strategies. Mulugeta and Hundie [43], Hagos [44], Yenesew and Masresha [45], and Duguma et al. [46] examined the impact of diversification strategies on food security only, ignoring nutrition security. Duguma et al. [46], Dinku [47], Akrasi et al. [48], Ayana et al. [49], Emeru et al. [50], and Washo et al. [51] in their part focused only on a single diversification option ignoring the combination of livelihood diversification. Tesfaye et al. [52], Hagos et al. [53], and Bekele [54] used ordinary least square (OLS), double-hurdled, ordered probit, and propensity score matching estimation approaches, which may be susceptible to endogeneity, self-selection bias, and insufficient in capturing alternative scenarios.

However, previous studies did not examine the relationship between livelihood diversification and food and nutrition security. They did not connect the effects of livelihood diversification to food and nutrition security. In this study, the researcher used a multinomial endogenous switching regression (MESR) model to address potential endogeneity and self-selection bias. As a result, this study addresses the gap in existing research by exploring the factors influencing livelihood diversification and its effects on food and nutrition security in rural households in Ethiopia.

## Methods

### Study area

**FIGURE 1:**
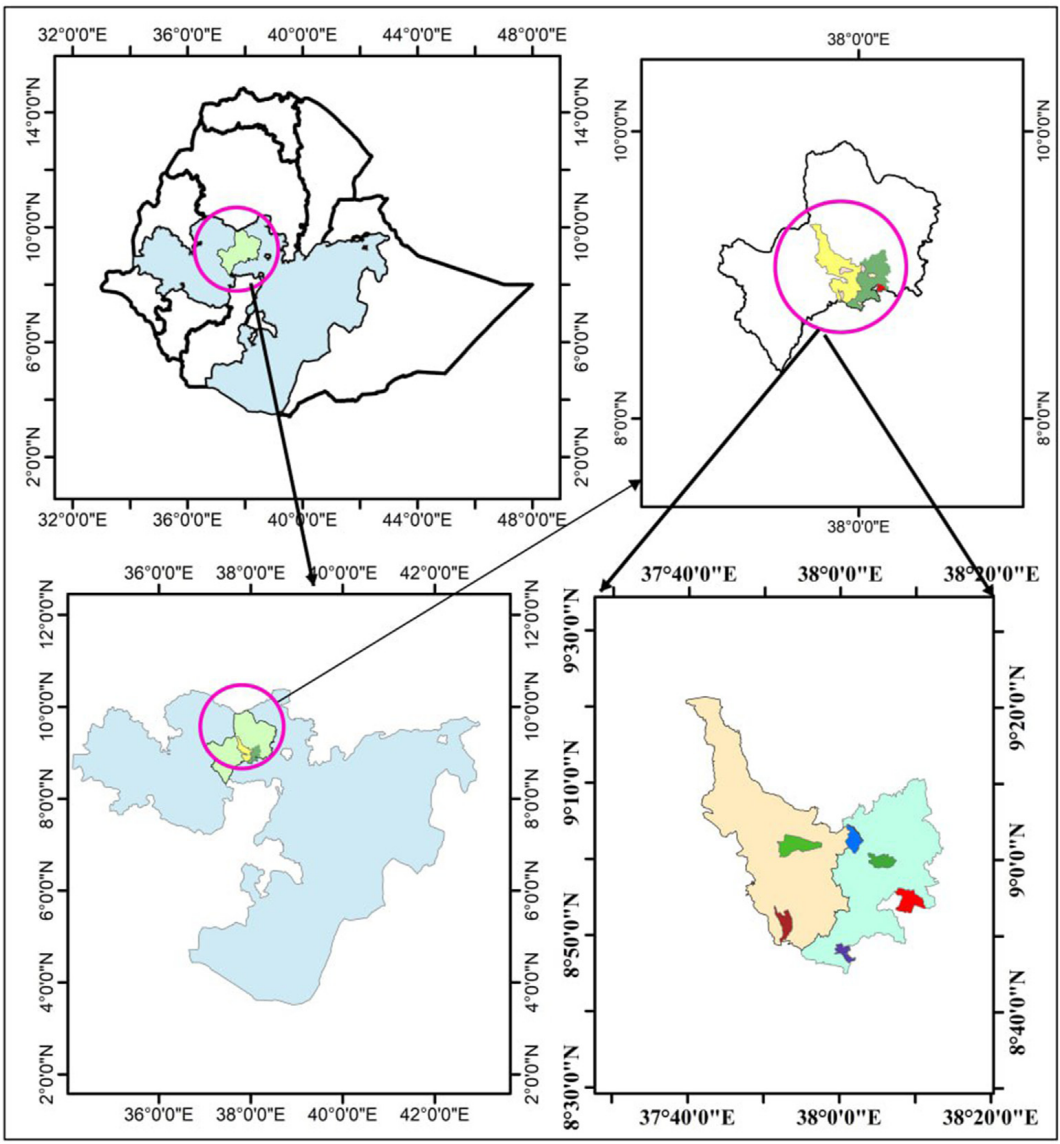
Map of the study area. HDDS, Household Dietary Diversity Score; HFIAS, Household Food Insecurity Access Score.

The study was conducted in Ambo and Dendi districts of West Shoa zone, Oromia Regional State, Ethiopia, located 114 km from Addis Ababa (Ethiopian capital). Geographically, the absolute location of the districts is between 8°40′0″N–9°200″N latitude and 37°40′0″E–38°20′0″E longitude (Figure 1). Agriculture serves as the primary economic activity, with ∼90% of the population relying on it for their livelihood. The predominant agricultural practices in the zone include crop production, plantation, animal husbandry, forestry, logging, and fishing. However, agriculture in the zone faces challenges such as moisture stress, soil erosion, limited arable land, shortage of draught power, high incidence of pests and diseases, livestock diseases, inadequate supply of agricultural inputs, and poor weed controlling. The Zone Report (2022) shows that certain kebeles in Ambo and Dendi districts are experiencing food and nutrition insecurity. To supplement their income, residents are engaging in off-farm and nonfarm activities.

### Data types and sources, and methods of data collection

The data for this study were gathered from primary and secondary sources. Secondary data were obtained from agricultural offices in the zone and sample districts. Primary data were collected from the sample farm households using a semistructured questionnaire administered by trained enumerators. A multistage sampling technique was employed to select the sample rural villages and farm households surveyed. First, Ambo and Dendi districts were purposively selected from a total of 33 districts in the zone based on their history of livelihood diversification and slight information on the rate of food insecurity of smallholder households. Second, 7 rural villages (3 from Ambo and 4 from Dandi) were randomly selected from the total of 34 rural villages in Ambo district and 48 in Dandi district. Third, after stratifying farm households into diversifiers and nondiversifiers, a total of 385 farm households (215 diversifiers and 170 nondiversifiers) were randomly and proportionally selected from the lists of household heads’ using a computer-generated random number table in the sample rural villages.

### Data processing and analysis

The study aimed to quantify the impact that livelihood diversification has on the food and nutrition security of smallholder farmers. The study employed quantitative methods of data analysis. With regard to quantitative data analysis, the survey data were coded, organized, and analyzed using descriptive statistics and econometric models. In particular, a multinomial logit model was utilized to analyze the factors that influence livelihood diversification strategies among rural farming households. Furthermore, the MESR model was employed to analyze the impacts of livelihood diversification on food and nutrition security for these households in the research area. The specific steps for the quantitative analysis of the gathered data are explained in the subsequent sections.

### Food and nutrition security measurements

There are various methods available for assessing food and nutrition insecurity, either directly or indirectly; the selection of the most suitable method(s) depends on the specific questions being asked, available resources, and time frames. It is important to share the lessons learned from different countries on how to enhance policy and program design by improving the conceptualization and measurement of food security. This knowledge should be systematically disseminated through networks of the researchers and practitioners [25]. The study assessed the household food and nutrition security status using the Household Food Insecurity Access Score (HFIAS) and the Household Dietary Diversity Score (DDS), both grounded in real-world conditions.

The study used the HFIAS to evaluate the food security status of households. The HFIAS is a continuous measure that assesses the level of food insecurity experienced by a household in the past 30 d. The total HFIAS score, which ranges from 0 to 27, indicates the degree of inadequate access to food. Despite potential limitations, the HFIAS is known to be easily understood and applicable in various settings. This measure reflects the household’s perception of their food situation, regardless of its nutritional content [54]. It implies that households’ experiences of food insecurity elicit predictable reactions and responses, which can be evaluated through a survey and condensed into a score.

Following Mango et al. [55], the HFIAS is computed as follows:

HFIAS (0–27) = summation of the frequency of occurrence during the past 30 d for the 9 food insecurity-related conditions

HFIAS(0−27)=Q1a∗F1+Q2a∗F2+Q3a∗F3+Q4a∗F4+Q5a∗F5+Q6a∗F6+Qa7∗F7+Q8a∗F8+Q9a∗F9

Categories of household according to HFIAS are shown in Table 1 [56,57].

**TABLE 1:** Categories of household according to HFIAS.

Categories	Description
Food-secure households	The household did not experience any of the food insecurity situation, or only had the experience of worrying about food, but rather infrequently ((*Q1* = 0) +(*Q1* = 1), *Q1a* = 1))
Mildly food-insecure households	The household worries about not having food to eat occasionally or frequently ((*Q1* = 1), *Q1a* = 2 or = 3)), and/or being unable to consume choice foods ((*Q2* = 1), *Q2a* = 1 or = 2 or = 3)), and/or having little variety of food ((*Q3* = 1), *Q3a* = 1)), and/or some food preferred to as unpalatable only on rare occasions ((*Q4* = 1), *Q4a* =1))
Moderately food-insecure household	The household consumes few varieties or unpalatable foods occasionally or frequently ((*Q3* = 1), *Q3a* = 2 or = 3)) + ((*Q4* = 1), *Q4a* = 2 or = 3)), and/or has begun to reduce the size or number of meals infrequently or occasionally ((*Q5* = 1), *Q5a* = 1 or = 2)) + ((*Q6* = 1), *Q6a* = 1 or = 2)) but did not experience any of the 3 extreme food insecurity situations (*Q7a–9a*)
Severely food-insecure households	The household has moved gradually to reducing the quantity of meal or number of meals most frequently ((*Q5* = 1), *Q5a* = 3)) + ((*Q6* = 1), *Q6a* = 3)), and/or experiencing the 3 most extreme situations such as “not having any food to eat,” “going to bed without eating any food,” or “going a whole day hungry,” even infrequently ((*Q7* = 1), *Q7a* = 1 or = 2 or = 3)) + ((*Q8* = 1), *Q8a* = 1 or = 2 or = 3)) + ((*Q9* = 1), *Q9a* = 1 or = 2or = 3))

Abbreviation: HFIAS, Household Food Insecurity Access Score.

The explanation on the categories of household food insecurity levels assessed based on the HFIAS module. The table shows that households with an HFIAS score of ≥1 are food-secure household; an HFIAS score of 2, 3, 4, 5, 6, 7, and 10 belonged to mildly food-insecure household; an HFIAS score of 8, 9, 11, 12, 13, 14, 16, and 17 belonged to moderate food-insecure household; households with an HFIAS score of 15, 18, 19, 20, 21, 22, 23, 24, 25, 26, and 27 are severely food-insecure household [56].

The household nutrition security status was measured using the DDS approach, which is based on 24-h food intake recalls of 12 food groups. Hoddinott and Yohannes [56] suggest that DDS is a meaningful measurement of nutritional status as it reflects household food access, correlates with food consumption, and is associated with improved birth weight and child anthropometric status. Research has shown that a higher DDS is linked to better nutrient adequacy in the diet [58,59]. The DDS assesses household nutrition security by determining if a family consumed ≥1 food item from each of the 12 categories in the previous 24 h [56]. The total score ranges from 0 to 12 based on the variety of food categories consumed.

### Multinomial logit model

The study utilized a multinomial logit selection model to analyze the determinants of decision-making process of farmers in choosing various livelihood diversification strategies within a random utility framework. Farmers were presented with the option to diversify their livelihoods by selecting single or combinations of available strategies. In this study, 4 livelihood diversification options were examined: farm only; farm and off-farm; farm and nonfarm; and farm, off-farm, and nonfarm. The multinomial logit approach was deemed suitable for modeling this decision process from an econometric perspective.

Mathematically, for a vector of interventions *j* (*j* = 1, …, *J*), the farmer *i* maximizes the expected utility function given by $W_{ij}$:

(1) $$W_{ij}=K_{i}\alpha +u_{ij}$$

where $K_{i}$ is observed exogenous variables affecting diversification; *u* represents unobserved characteristics that capture an individual’s tendency to diversify; and *α* stands for a vector of parameters to be recovered in the first stage estimation.

The farmer’s utility from choosing a combination of diversification options, $W_{ij}$, is not observable, but the choice will be. The decision rule is that farmer *i* chooses a combination of diversification option *j* if $K_{ij}$ gives him the maximum possible utility. This implies that the farmer will choose the combination of diversification option, *j*, in preference to choose any other combination of strategies, *s*, if:

(2) $$W=\left\{ \begin{array}{c} 1\;\text{iff}\;W_{i1}>\mathrm{Max}\;W_{is}\vee \gamma_{i1}\langle 0\\ s\neq 1\\ .\\ .\\ .\\ J\;\text{iff}\;W_{ij}>\mathrm{Max}\;W_{is}\vee \gamma_{iJ}\langle 0\\ s\neq J \end{array}\right.\;\text{for}\;\text{all}\;s\neq J$$

Equation *2* implies that the ith farmer will choose a combination of strategies to maximize his expected food and nutrition security if it provides greater expected food and nutrition security than any other package, that is, if $\gamma_{ij}=\max\left(W_{is}-W_{ij}\right)<0\text{,}$ s≠J.

As per Equation *1*, it is assumed that the covariate vector $K_{i}$ is uncorrelated with the idiosyncratic unobserved stochastic component *e*_*ij*_. Under the assumption that $e_{ij}$ are independent and identically Gumbel distributed [that is, under the Independence of Irrelevant Alternatives (IIA) hypothesis], selection model *2* leads to a multinomial logit model [60], where the probability of choosing strategy j ($P_{ij}$) is:

$$P_{ij}=\mathrm{Pr}\left(\gamma_{i1}\langle 0|K_{i}\right)=\frac{\exp\;\left(K_{i}\alpha_{j}\right)}{\sum_{s=1}^{j}\exp\;\left(K_{i}\alpha_{s}\right)}\;\text{.}$$

### Multinomial endogenous switching regression model

The study estimated the impact livelihood diversification on food and nutrition security of farm households using MESR, which captures selection bias and heterogeneity because of observable and unobservable factors. A 2-stage framework was applied to evaluate the impact of livelihood diversification on food and nutrition security, with detailed explanations of the econometric model employed given below.

In the second stage of MESR, the analysis examines the relationship between livelihood diversification strategies and food and nutrition security outcomes, incorporating an explanatory variable (*Z*) related to assets. Each diversification strategy is analyzed individually, with farming only (*J* = 1) serving as the baseline category. The strategies considered are farming only (*j* = 1), farming plus off-farming (*j* = 2), farming plus nonfarming plus off-farming (*j* = 3), and farming plus both off-farming and nonfarming activities (*j* = 4). To account for potential unobservable factors influencing farmers’ decisions on diversification or nondiversification that could be connected to the desired results, an MESR method is employed in this study. This approach captures both self-selection bias and the interactions between different diversification choices, as noted by Biru et al. [60] and Ding and Abdulai [61]. The MESR model is chosen for its ability to tackle challenges including selection bias, endogeneity, and counterfactuals in impact evaluation estimation.

On the basis of Danso-Abbeam and Baiyegunhi [62] and Kumar et al. [63], it is assumed that farmer *i* aims to maximize their net returns, *U*_*i*_, by comparing the earnings from various livelihood diversification options *J* (*J* = 1, 2, 3, 4) with any other approach, *m*. If *U*_*ij*_ > *U*_*im*_; *m* ≠ *J*, household *i* will choose package *J* over any other option, package *m*. If the utility of the chosen combination is higher than that of other packages, it is assumed that the households followed the specified livelihood diversification plan. However, as per Biru et al. [60], Ding and Abdulai [61], Belay and Mengiste [64], and Ahmed [65], the household’s expected utility *(∪*_*ij*_∗) from participating in livelihood diversification strategies *J* can be defined as:

(3) $$\cup_{ij}*X_{i}\beta_{j+\varepsilon_{ij}}$$

${\cup *}_{ij}$ is a latent variable that, based on both observed and unobserved factors, defines the projected net advantages that the household hopes to obtain from the lifestyle diversification combination option *J*. An error term called ε_ik_ accounts for unobserved qualities, whereas *X*_*i*_ provides a vector containing the covariate or exogenous factors observed. Among the various combinations of livelihood diversification techniques that are provided, let *D* be the index of household options. This way, *D* can be expressed as:

**Figure:**
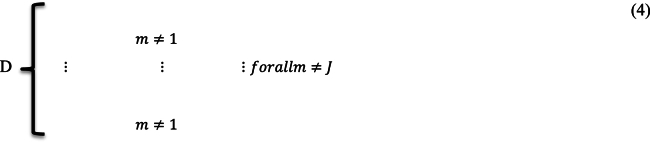

In Equation *4*, the index function indicates that the *i*th household will engage in livelihood diversification strategy *J* if they receive or anticipate a greater benefit or net income from package *J* compared with any other package m ≠ *J*. As illustrated in Table 1, the reference category, which does not diversify into any of the provided livelihood diversification strategies, is denoted by *D* = 1 (Regime 1), whereas the remaining alternative combinations are denoted by *D* = 2, 3, 4 … *J*, signifying diversification using ≥1 combination of the given strategies. For every possible regime, the following is the link between the set of exogenous factors *H* and the result variables:

**Figure:**
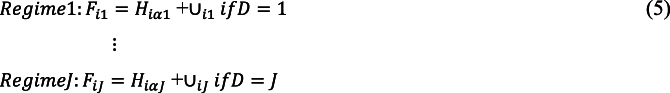

where F s are the outcome variables that represent nutrition security (DDs), or food security (HFIAS) of the ith households at different diversification option *D* (*D* = 1, 2, 3 … *J*), and *u*s are error terms. If the εs in Equation *3* and us in Equation *4* are not independent, the estimation of Equation *5* using linear regression, that is OLS, will be biased. Hence, a consistent estimation of $\alpha_{J}$ requires inclusion of the selection terms of the alternatives in Equation 6. The linearity assumption of Dubin and McFadden’s [66] model can be used to solve this:

(6) $$\in \left(\frac{\cup_{ij}}{\varepsilon_{i1}}\cdots \cdots \cdots \varepsilon_{iJ}\right)=\sigma_{J}\sum_{m\neq J}^{J}r_{J}\;\sum_{m=1}^{J}rJ=0$$

where $\sigma_{J}$ is the covariance between the error terms *ε* in Equation *1* and *u* in Equation *3*, ωs are the error terms with a zero expected value, and $\lambda_{J}$ is the inverse Mills ratio derived from the estimated probabilities in Equation *2*, which is computed as:

(7) $$\lambda_{J}\;\sum_{m\neq J}^{J}\rho_{J\left[\frac{\rho_{I}\;\ln\;\rho_{I}}{1-\rho_{I}}+\ln\left(\rho im\right)\right]}$$

The correlation coefficient *ρ* represents the relationship between the error terms εs, *u*s, and ωs in the selection model’s nonlinear functional form. This form is effective in estimating the characteristics of a model for MESR, even when the regression in the outcome and selection equations is similar. Biru et al. [60], Ding and Abdulai [61], and Chamberlain and Griliches [67] demonstrated that instrumental variables are not always required for identifying a system of equations. Although Biru et al. [60], Ding and Abdulai [61], and Ahmed [65] recommend including selection instruments in the alternative choice model specified in Equation *3*, for consistent parameter estimates in a multinomial treatment effect model, it is not strictly necessary to include an instrument in the selection equation. The exclusion restriction test, which involves removing factors that alter the participation equation but not the outcome equation, is recommended by Kassegn and Endris [28], Tesfaye et al. [68], and Asmare et al. [69] as a means of confirming the validity of the switching regression model.

### Average treatment effect estimation

Analyzing the treated (ATU^2^) and untreated (ATU^3^) groups allows us to calculate the average treatment effects using the MESR model framework. This involves comparing the expected outcome values of the treated (*D* = 2, 3, 4, ... *J*) and untreated (*D* = 1) groups in both real and counterfactual scenarios. Following the methodologies outlined in the studies by Biru et al. [60], Belay and Mengiste [64], Ahmed [65], and Zegeye et al. [70], we can determine the average treatment effect on the treated (ATT) and untreated (ATU) by comparing the expected outcomes with and without diversification.

Diversifiers with diversification of *D* combination (actual expectation observed in the sample):

**Figure:**
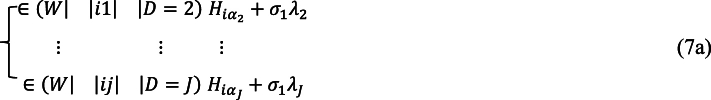

**Figure:**
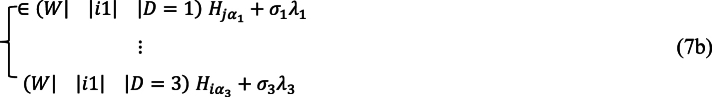

Diversifiers had decided not to diversify (counterfactual expectation that are detected):

**Figure:**
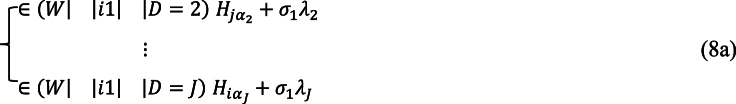

**Figure:**
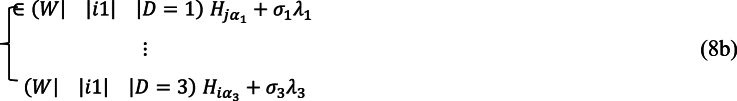

Next, the ATT is calculated by comparing the results of Equations *7a* and *8a*. The diversifiers in the second group of the specified diversification strategy, *D* = 2, have their ATT estimated by comparing the initial equations in *7a* and *8a*, which is equivalent to:

(9) $$\text{ATT}\in \left[\frac{W_{i2}}{D}=2\right]-\in \lfloor \frac{W_{j1}}{D}=2\rfloor =H\left(\alpha_{2}-\alpha_{1}\right)+\lambda \left(\sigma_{2}-\sigma_{1}\right)\text{.}$$

Similarly, ATU is calculated by comparing Equations *7a* and *8b*. For instance, the ATU for nondiversifiers in the second diversification strategy combination is determined by subtracting the initial equations in *7b* and *8b*. This calculation is represented as:

(10) $$ATU\in \left[\frac{W_{i21}}{D}=1\right]-\in \lfloor \frac{W_{j2}}{D}=1\rfloor =H\left(\alpha_{2}-\alpha_{1}\right)+\lambda \left(\sigma_{2}-\sigma_{1}\right)\text{.}$$

### Reliability test

The HFIAS revealed Cronbach’s alpha of 0.7332, indicating very high reliability [71]. Among the 9 food access indicator questions provided, a majority of respondents were from households residing in the Dendi woreda where livelihood diversification strategies were implemented.

## Results

The study examined 215 farm households that diversified their livelihoods and 170 that did not diversify, assessing the effects of diversification strategies on food and nutrition security.

### Sample farm households by livelihood diversification strategies

More than half (56%) of the farming households were able to diversify their livelihoods through off-farm, nonfarm, or combined activities. Conversely, 44% of the households could not to diversify their livelihoods, often lacking the means to engage in any form of livelihood diversification beyond agricultural activities (Table 2).

**TABLE 2:** Livelihood diversification strategies and the distribution of sample households (385).

Strategies	Abbreviations	Frequency	Percent
On-farm only	F_1_N_0_O_0_	170	44.2
On-farm and off-farm	F_1_O_1_N_0_	84	21.8
On-farm and nonfarm	F_1_O_0_N_1_	77	20.0
Joint diversification of 3 strategies	F_1_N_1_O_1_	54	14.0
Total		385	100

### Descriptive statistics of household characteristics

A 1-way analysis of variance F-test and χ^2^-test were employed to examine variations in rural households’ livelihood diversification among strategies rural households’ based on their mean scores for continuous and dummy predictor variables. The F-test analysis revealed significant differences in mean values among rural households across 4 livelihood diversification strategies concerning age, education, family size, distance to the nearest market, farm experience, and total annual income. The results indicate that farm households headed by young, educated family members living close to markets, with more farm experience, and lower annual incomes are more likely to adopt diversified livelihood strategies compared with other households. Results of the χ^2^-test for categorical variables show that farm household, access to extension services, financial capital, landownership, livelihood training, social norms, policy, and information on climate significantly influence livelihood diversification at varying significance levels (Table 3).

**TABLE 3:** Descriptive statistical results for sample households’ characteristics.

Continuous variables	F_1_O_0_N_0_	F_1_O_0_N_1_	F_1_O_1_N_0_	F_1_O_1_N_1_	Total	F-test
Age (y)	41.6 (14.9)	37.6 (8.3)	37.8 (11.9)	38.0 (9.9)	39.5 (12.6)	12.61∗∗
Education (y)	3.9 (3.7)	5.6 (2.9)	5.6 (3.4)	4.4 (3.0)	4.7 (3.5)	7.2∗∗∗
Family size (*n*)	5.1 (3.0)	4.6 (2.2)	4.7 (2.5)	5.0 (3.1)	4.9 (2.8)	22.0∗∗∗
Farm size (ha)	1.6 (1.0)	1.7 (1.3)	1.6 (1.1)	1.6 (1.1)	1.6 (1.1)	0.4
Distance to market (km)	3.8 (2.3)	3.2 (2.1)	3.3 (2.5)	2.8 (2.2)	3.5 (2.3)	3.5∗∗
Annual income (Birr)	13,788.5 (15,431.4)	17,355.0 (27,496.8)	109,708 (4373.6)	10,736.1 (3850.8)	13,575 (16,724.1)	2.6∗
Livestock owned (TLU)	4.3 (4.0)	4.1 (4.1)	4.6 (4.1)	5.0 (4.5)	4.4 (4.1)	0.6
Farming experience	22 (9.26)	22.09 (8.65)	22.50 (8.8)	21.06 (8.06)	22.16 (8.05)	8.8∗∗∗

Categorical variables	F_1_O_0_N_0_	F_1_O_0_N_1_	F_1_O_1_N_0_	F_1_O_1_N_1_	Total	χ^2^ value

Sex (male)	155 (45.6)	74 (21.7)	69 (20.2)	43 (12.6)	341 (100)	5.5
Credit (yes)	43 (37.1)	33 (28.5)	22 (19.0)	18 (15.5)	141 (100)	5.9
Extension (yes)	66 (46.8)	12 (8.5)	23 (16.3)	40 (28.4)	141 (100)	52.6∗∗∗
Working capital (yes)	156 (40.52)	75 (21.7)	69 (17.92)	42 (10.91)	341 (88.57)	3.99∗∗∗
Land ownership (yes)	145 (37.66)	48 (12.47)	50 (12.99)	32 (8.31)	275 (71.43)	26.45∗∗∗
Livelihood training (yes)	133 (34.55)	58 (15.06)	54 (14.03)	5 (1.3)	250 (69.94)	83.02∗∗
Social norms (yes)	151 (39.22)	66 (26.0)	66 (17.14)	62 (16.10)	314 (81.56)	12.01∗∗∗
Policy	30 (7.79)	51 (13.51)	47 (12.21)	31 (8.05)	160 (41.56)	74.51∗∗∗
Climate variability (yes)	18 (4.68)	37 (9.61)	36 (20.8)	33 (5.8)	261 (67.79)	73.35∗∗∗

∗Significant at *P* < 0.1; ∗∗significant at *P* < 0.05; ∗∗∗significant at *P* < 0.01.

### Determinants of choice of livelihood diversification strategies in Ethiopia

Various diagnostic tests were conducted to accurately estimate the model. The test for IIA assumption using the Hausman approach showed that the null hypothesis H0: the difference in coefficients is not systematic could not be rejected (Prob > χ^2^ = 0.3244), indicating the appropriateness of the multinominal logit model. The Wald test results supported the alternative hypothesis that all regression coefficients are collectively different from zero (*P* > χ^2^ = 0.000). Robust regression was used in the estimation process to address concerns regarding heteroskedasticity and non-normality of the error terms (Table 4).

**TABLE 4:** Determinants of livelihood diversification strategies by smallholder farming households.

Variable	Livelihood strategies adopted by sample households					
	Farm+ off-farm		Farm + nonfarm		Farm +off-farm+ nonfarm	
	Dx/dy	Coef. (Robust Se)	Dx/dy	Coef. (Robust Se)	Dx/dy	Coef.(Robust Se)
Sex	0.059∗∗∗	0.356 (0.154)	–0.013	0.057 (0.1478)	0.004∗	0.359 (0.210)
Age	–0.002	0.020 (0.013)	–0.001	–0.171 (0.144)	–0.005∗	–0.045 (0.025)
Family size	–0.059	–0.019 (0.068)	0.000	0.003 (.065)	0.004∗∗∗	0.258 (0.108)
Education	–0.021	–0.236 (0.181)	0.049∗∗∗	–0.330 (0.173)	0.001	–0.075 (0.237)
Farm experience	0.005∗∗	0.044 (0.020)	0.004∗	0.036 (0.020)	–0.000	–0.084 (0.035)
Social norms	–0.156∗∗∗	–1.356 (0.428)	–0.137∗∗∗	–0.130 (1.205)	–0.035∗∗∗	–2.757 (0.594)
Land ownership	–0.207∗∗∗	–1.558 (0.395)	–0.123∗∗∗	–1.154 (0.420)	0.001	–0.575 (0.582)
Working capital	0.005	0.017 (0.523)	–0.0002	0.0284 (0.500)	0.026∗∗∗	1.633 (0.538)
Livestock	0.000	0.019 (0.043)	0.005	0.037 (0.042)	0.001∗∗∗	0.094 (0.054)
Credit use	0.114∗∗	0.699 (0.342)	0.001	0.225 (0.356)	–0.011	–0.448 (0.545)
Extension access	––0.178∗∗∗	–0.989 (0.46)	0.173	–0.180 (0.399)	0.044∗∗∗	2.396 (0.548)
Policy	0.217∗∗∗	1.646 (0.498)	0.177∗∗∗	1.400 (0.481)	–0.043∗∗∗	–1.848 (0.641)
Climate	0.058	0.814 (0.538)	0.133∗∗∗	1.069 (0.546)	0.080∗∗∗	5.329 (0.841)
Livelihood	–0.036	–0.465 (0.377)	–0.033	–0.449 (0.373)	–0.080∗∗∗	–5.123 (0..635)
Distance to district	––0.016∗∗∗	–0.130 (0.073)	–0.020	0.100 (0.075)	–0.002∗∗∗	–0.219 (0.098)

Dependent variable: livelihood diversification.

Reference category: farm alone.

Number of observation: 385.

Log pseudo likelihood model: 340.566.

LRch^i2^(51) = 213.09; Prob >chi^2^: 0.00.

∗∗∗, ∗∗, and ∗ denote the significance level at 1%, 5%, and 10%, respectively, and SEs in parentheses.

The coefficient of the sex of the rural household’ head was a positive and statistically significant at <1% and 5% levels, indicating a positive impact on diversification activities. The marginal effects of the model show that, all else being equal, male-headed households are 5.9% more likely to adopt a farm plus off-farm diversification strategy and 0.4% more likely to adopt a farm plus off-farm plus nonfarm strategy compared with female-headed households. Conversely, the age of the household head has a significant negative effect on the choice of farm plus off-farm plus nonfarm activities at <1% level. The marginal effect suggests that as household heads age, the probability of selecting farm plus off-farm plus nonfarm activities decreases by 0.5%, indicating that younger farmers are more likely to engage in diversified livelihoods compared with older farmers in the study areas.

The study found that household size had a significant positive impact on the choice of on-farm plus off-farm plus nonfarm diversification activities at a significance level of <1%. The marginal effect results showed that, holding other variables constant, the likelihood of adopting these livelihood strategies increased by 0.4% for each additional household member compared with those engaged only in on-farm activities. Additionally, the education level of the household head significantly influenced the household’s diversification into farm plus nonfarm activities at a significant level of <1%. Holding other factors constant, households with higher levels of education were 0.4% more likely to choose farm plus nonfarm livelihood diversification strategies compared with noneducated households. Furthermore, the results suggested that farming experience had a positive and significant relationship with livelihood diversification at significance levels of <5% and 10%, respectively. The marginal effects of farming experience indicated that, although other factors remained constant, the probability of adopting livelihood diversification strategies involving farming plus off-farming plus nonfarming activities increased by 0.5% and 4.4%, respectively, for each additional year of farming experience.

The study indicated that social status of household head has a significant negative influence on farmers’ decisions to diversify their livelihoods through various combinations of activities at a statistically significant level of <1%. The marginal effects of social status indicated that, holding other factors constant, the probability of farmers opting for livelihood strategies involving farming in along with off-farm activities, on-farm and nonfarm activities, and a combination of farming plus off-farm plus nonfarm activities decreased by 15.6%, 13.7%, and 3.5%, respectively, with an increase in the household’s social status. The study also found that owning land in the farming sector significantly decreased the likelihood of farmers engaging in farming alongside off-farm and nonfarm activities at a significance level of <1%. The marginal effects indicated that owning land could reduce the probability of farmers participating in farming combined with off-farm and nonfarm activities by ∼20.7% and 12.3%, respectively, although controlling the other variables.

The study showed that the working capital of the household head had a positive and significant influence on farmers’ decisions to diversify their livelihoods into farm, off-farm, and nonfarm activities at a significant level of <1%. The marginal effect indicated that as the financial capital of the household head increased, the likelihood of rural households participating in the diversification activities also increased by 2.6%, although keeping other variables constant. The study revealed a statistically significant and positive association between households’ livelihood diversification choice to farm plus nonfarm and off-farm and tropical livestock unit (TLU) at a significant level of <1%. Access to credit service has a substantial positive effect on diversifying livelihood strategies in both farming and off-farming activities at significance level of <5%. Holding all other variables constant, the likelihood of smallholder households engaging in both farming and off-farming activities increases by 11.4% for every additional unit of credit access. The study also found that accees to extension services have a negative and significant relationship with the likelihood of diversifying into farming and off-farm livelihood strategies within agricultural activities at significance level <1%. However, extension services are positively and significantly associated with the likelihood of diversifying into farming, off-farm, and nonfarm activities at the same significance level. The marginal effects of access to extension services show that, all else being equal, the probability of households participating in farming plus off-farming, and farming plus off-farming plus nonfarming livelihood diversification activities decreases and increases by 17.8% and 4.4%, respectively, when they have access to extension services.

The findings suggest that government policy plays a significant role in influencing the choice to diversify livelihoods, with noticeable impacts seen at a significance level below 1%. Specifically, households are 21.7% more likely to participate in farming and off-farm activities when they receive guidance from government policies. On the other hand, the probability of engaging in farming and nonfarming activities decreases by 17.7% with government policy direction. Additionally, the likelihood of households participating in farming, off-farm, and nonfarm activities decreases by 4.3% when influenced by government policy guidance.

The study revealed that climate variability information has a significant impact on the diversification of livelihood strategies in farm plus nonfarm, and farm plus off-farm plus nonfarm activities, with at significance level of <1%. The marginal effects revealed that for each additional instance of climate variability experienced by the household head, there was a 13.3% increase in farm plus off-farm activities and an 8.0% increase in farm plus nonfarm activities. The study showed that, livelihood training was found to have a negative and significant effect on the decision to engage in a combination of on-farm, nonfarm, and off-farm activities, with a significance level of <1%. The marginal effect indicated that households with access to livelihood training had an 8.0% lower probability of diversifying their livelihood activities compared with those without training. Moreover, the distance from the nearest market had a negative and significant impact on households’ choices of on-farm plus off-farm and farm plus off-farm plus nonfarm activities at a significance level of <1%. The marginal effect showed that, holding other factors constant, a 1-km increase in market distance decreased the likelihood of choosing on-farm plus off-farm and farm plus nonfarm plus off-farm activities by 1.6% and 0.2%, respectively.

### Average treatment and heterogeneity effects estimation-impact of livelihood diversifcation strategies on food and nutrition security

MESR model was employed to conduct a thorough evaluation of the impact of livelihood diversification strategies on food and nutrition security among smallholder farm households in the study area. Results are presented in Table 5.

**TABLE 5:** Treatment and heterogeneity effect for multinomial endogenous switching regression.

Outcome variable	LDS option (D)	Decision stage		Average treatment effect (ATT)
		To diversify (D = 2, 3, 4)	Not to diversify (D = 1)	
HFIAS	F_1_O_0_N_1_	0.47 (0.06)	0.09 (0.11)	0.75 (0.09)∗∗∗
	F_1_O_1_N_0,_	0.44 (0.07)	0.28 (0.13)	0.71 (0.12)∗∗∗
	F_1_O_1_N_1_	–0.04 (0.06)	0.77 (0.14)	0.33 (0.10)
HDDS	F_1_O_0_N_1_	2.39 (0.06)	1.97 (0.04)	0.42 (0.07)∗∗∗
	F_1_O_1_N_0,_	2.19 (0.07)	1.97 (0.04)	0.22 (0.08)∗∗∗
	F_1_O_1_N_1_	2.12 (0.07)	1.97 (0.04)	0.15 (0.08)∗

Heterogeneity effects		HE_1_	HE_0_	TH = ATT – ATU

HFIAS	F_1_O_0_N_1_	–0.56 (0.12)∗∗∗	0.11 (0.10)	–0.66 (0.09)∗∗∗
	F_1_O_1_N_0_	–0.67 (0.12)∗∗∗	0.24 (0.11)∗∗	0.43 (0.12)∗∗∗
	F_1_O_1_N_1_	0.32 (0.14)∗∗	0.08 (0.12)	0.24 (0.12)∗∗
HDDS	F_1_O_0_N_1_	–0.20 (0.09)∗∗	0.23 (0.07)∗∗∗	–0.43 (0.09)∗∗∗
	F_1_O_1_N_0_	–0.10 (0.11)	0.07 (0.07)	0.16 (0.11)
	F_1_O_1_N_1_	0.09 (0.14)	0.32 (0.08)∗∗∗	–0.23 (0.12)∗

HDDS, Household Dietary Diversity Score; HFIAS, Household Food Insecurity Access Score; LDS, livelihood diversification strategies.

∗∗∗, ∗∗, and ∗ denote the significance level at less than 1%,5% and 10% respectively.

The estimated results of the ATT indicate that, engaging in both farm and off-farm livelihood diversification strategies enhance farm households’ food security by 75% compared with households that do not diversify. The finding is statistical significance at a significant level of <1%. The findings suggest that households engaging in farming combined with nonfarming livelihood diversification strategies experience a 71% improvement in their food security status compared with those that do not. This result is also statistically significant at a probability level of <1%, indicating a strong relationship between these diversification strategies and enhanced food security. According to the average treatment effect estimation, combining farming with off-farming and nonfarming livelihood diversification strategies has no significant impact on-farm households’ nutritional security status.

The average treatment effect estimation also shows that engaging in farming plus off-farming livelihood diversification strategies increases farm households’ nutrition security by 42%, whereas farming plus nonfarming strategies result in a 22% improvement compared with nondiversifier households, with statistical significance level at <1%. Furthermore, combining farming with off-farming and nonfarming livelihood diversification strategies leads to a 15% enhancement in nutrition security status for farm households compared with nondiversifier households, with statistical significance level at <1% (Table 5).

The results in Table 5 reveal significant transitional heterogeneity effects (TH) for nondiversifiers (HE0) and diversifiers (HE1), indicating notable differences between the 2 groups. Diversified households exhibit greater food security than their nondiversified counterparts, as indicated by the negative HE0 values. Conversely, the negative HE1 values suggest that nondiversified households face less food security. Furthermore, transitional heterogeneity has a positive and statistically significant impact on livelihood diversification. Farm households that engage in off-farm and nonfarm activities alongside with their agricultural practices demonstrate a significantly positive difference compared with those who do not diversify into these areas.

## Discussion

Our findings have several policy implications. The involvement of smallholder farmers in livelihood helps to reduce poverty, nutrition security, food insecurity, and unemployment. The result of this study has proven that the involvement of livelihood diversification by smallholder farmers had a positive impact on food and nutrition security. This is because smallholder farmers are involved in livelihood diversification, and they are able to produce more for consumption and sell more to generate income.

The gender of the household head positively and significantly influenced the engagement of livelihood diversification strategies, especially in farm plus off-farm and nonfarm activities. Households headed by males are more likely to engage in a variety of livelihood activities compared with those headed by females. In Ethiopia, female-headed households are tend to be less inclined to diversify their livelihood strategies, possibly because of cultural norms that assign them responsibilities such as childcare and household chores, which limit their ability to engage in other activities. This finding aligns with the research conducted by Ambachew and Ermiyas [72], Mulugeta Habtewold and Heshmati [73], and Alamneh et al. [74], which emphasize the challenges faced by Ethiopian female-headed households in economic activities. This contrast with the findings of Nasai [75] and Yenesew [76], which may suggest different dynamics regarding livelihood diversification.

As rural household heads age, their ability to participate in various livelihood activities may be constrained. This limitation is likely from older individuals facing challenges in diversifying their income sources and relying on a few strategies to support their families [77]. According to Kassie and Fellizarjr [78], younger individuals have greater opportunities to diversify their livelihood activities compared with older farmers, particularly because of limited access to land resulting from the expansion of construction and service industries in Ethiopia. As noted by Asfir [79], the age of the household head in rural areas influences their engagement in survival strategies. Elderly smallholders, who are often more established and experienced in farming, are less inclined to adopt new ideas and information, leading to reluctance to diversify their means of survival. However, it is important to recognize that the age of the household head can also positively impact livelihood diversification strategies [80,81] as skills and knowledge often improve with age, facilitating diversification.

The study showed that household size positively and significantly affect the choice of on-farm plus off-farm plus nonfarm livelihood diversification activities. Large families are more likely to engage in a combination of livelihood, because they can provide more household labor and meet the corresponding demand for food. The positive association aligns with the findings of Emeru et al. [50], Tamerat and Borah [82], Abera et al. [83], and Bird et al. [84], whereas it contradicts with the study by Gebru et al. [85], which suggested that a large household size does not mean all the household members contribute as productive labor.

The study showed that education level positively correlates with livelihood diversification among smallholder households encompassing farm plus nonfarm plus off-farm, and ability to access and evaluate opportunities and market prospects. Education equips farmers with the necessarily skills to effectively seek and utilize information effectively, making them more adept at leveraging technology and resources for diversification their livelihood options. The positive impact of education on livelihood diversification is seen in line with existing literature [50,85,86]. Additionally, the findings indicated a significant positive relationship between farming experience and household livelihood diversification strategies, particularly in farm plus off-farm and farm plus nonfarm activities. Farming experience foster the skills, knowledge, and confidence needed for successful livelihood diversification. Enhancing individual resilience and contributing to the sustainability and stability of economic of rural communities. By leveraging their experience, farmers can create diverse income streams which mitigate risks and adapt to changing conditions. These findings are consistent with previous studies by Duguma et al. [46].

The study also indicated that the social status of the household head has a negative and significant impact on farmers’ decisions to diversify their livelihoods into various combinations of activities attributed to the perception that smallholders engaging in nonfarm activities are going against traditional agricultural practices. These findings contrast with a study by Duguma et al. [46], Pokharel et al. [87], and Sisay [88], which found that social status positively influenced smallholder households’ livelihood diversification strategies. Furthermore, the study showed that land ownership had a significant negative impact on farmers’ likelihood of engaging in a combination of farm and off-farm or nonfarm activities. Previously studies conducted by Shekuru et al. [81], Tamerat and Borah [82], Anshiso and Shiferaw [89], and Beyene et al. [90] also identified that the extent of land ownership by rural families had an inversely and detrimental influence on livelihood diversification measures beyond agriculture. Conversely, according to Kebede and Singh [91], the extent of land ownership by rural families positively impacts income diversification measures outside agriculture because rural families with larger holdings can supplement their income by working as casual laborers to smooth out their farm operations.

Working capital emerged as a crucial factor enabling smallholder farm households to diversify their livelihoods by engaging in off-farm and nonfarm activities. Household heads with higher working capital were more likely to pursue diversification strategies, such as livestock rearing, small-scale trading, and nonfarm businesses, in addition to traditional farming. This diversification helps reduce reliance on a single income source and manage risks associated with agriculture, such as climate variations and market fluctuations. Financial resources also enable farmers to invest in inputs and technology, improving productivity and profitability. Furthermore, financial capital can support education and skill development, empowering farmers to effectively engage in nonfarming activities and enhance their overall income opportunities. This finding is consistent with the findings of Habib [92].

The study found a significant and positive correlation between rural household livelihood diversification in both farm and nonfarm activities and off-farm activities and TLUs. This suggests that rural families with larger TLUs are more likely to diversify their livelihood strategies beyond agriculture compared with rural households with smaller TLUs. These findings align with earlier studies conducted by Abera et al. [83], Girma [93], Shikur et al. [94], and Dirribsa and Tassew [95]. Access to credit was positively associated with livelihood diversification to farm plus off-farm among farmers underscoring the intricate relationship between financial resources and economic behavior. Access to credit can have a complex relationship with livelihood diversification activities. On one hand, credit can facilitate diversification by providing the financial resources needed to invest in new ventures. In this study, the results suggest that households do rely on financial services for livelihood diversification; rather, they are able to finance through directly available resources. This aligns with previous studies by Debele and Desta [35], Anshiso and Shiferaw [89], Bayu and Gondar [96], and Mentamo and Geda [97], which all showed a positive impact of credit access on diversifying livelihoods.

The study found a negative and significant correlation between access to extension services and the likelihood of diversifying into farming and off-farm livelihood activities. However, there was a positive and significant association between access to extension services and the likelihood of diversifying into farm, off-farm, and nonfarm activities at the same level of significance. This suggests that although extension services can provide valuable support livelihood diversification, they can also inadvertently limit livelihood diversification focusing too narrowly on traditional practices, unrelated government agenda, creating dependency, or failing to introduce alternative opportunities. Extension services play crucial role in agricultural development and rural livelihood diversification, but they can hinder diversification efforts because of various factors such as emphasis on traditional methods, limited understanding of livelihood options, inadequate training for extension workers, biases in resource allocation, lack of personalized support, market constraints, and social and cultural barriers. These findings are consistent with previous studies by Hagos [44], Duguma et al. [46], Bird et al. [84], and Wudil et al. [98].

The study revealed that government policies significant impact farmer’ decisions into diversify their livelihoods into farm plus off-farm, farm plus nonfarm, and farm plus off-farm plus nonfarm at a significance level at <1%. This underscores the crucial role of consistent government policies in assisting smallholders in effectively diversifying their income sources. Such policies can greatly influence the capacity of smallholder farmers to broaden their income streams. Through the provision of support services, market access, financial aid, and education, governments can establish an enabling environment that fosters the resilience and economic sustainability of smallholder farmers. Diversification not only aids in income stabilization but also contributes to food security and the adoption of sustainable agricultural practices. This is in line with the finding of Alemayehu et al. [99].

The study showed a positive correlation between household livelihood diversification strategies to farm plus off-farm and the availability of climate variability information to the household. Access to climate information enables smallholder farmers to diversify their livelihood strategies and adapt to environmental changes. Informed smallholders are better equipped to adjust their practices, leading to improved resilience, sustainable agriculture, and economic outcomes for rural communities reliant on agriculture. Similar conclusions have been drawn by other studies [[100], [101], [102]]. Engagement in livelihood training had a positive impact on households’ decision to engage in a mix of on-farm, nonfarm, and off-farm activities, indicating that households that received income-generating training were more likely to broaden their livelihood activities compared with those who did not receive training. The training assists farmers in enhancing their expertise and understanding, allowing them to pursue additional income-generating prospects. This finding is consistent with research conducted by Asfir [79].

The study found that the distance to the market has a negative impact on the diversification of livelihoods among smallholder farmers. Households situated further away from the market are less inclined to engage in a variety of income-generating activities, including farming, off-farm work, and nonfarm activities. On the other hand, those closer to market have more opportunities to participate in different sources of income diversification. This discrepancy can be attributed to the difficulties faced by households living far from the market in transporting their produce, which limits their ability to explore diverse income opportunities. Previous research has also highlighted that households near market areas have greater potential for income diversification compared with those residing further away. However, conflicting findings exist in the literature, with some studies suggesting that households that are further away from the market center struggle to diversify their income through nonfarm [29,39,74,103,104].

The estimated results of the ATT suggest that participating in both farm and off-farm livelihood diversification strategies improves food security for farm households compared with those who do not diversify. Diversifying livelihood activities can boost households’ nutritional intake, increase their resilience to economic and environmental challenges, and support sustainability, ultimately enhancing food and nutrition security. The findings are consistent with the studies carried out by Taye et al. [27], Abebe [31], Duguma et al. [46], Dinku [47], Sisay [88], Mahama and Nkegbe [105], Afodu et al. [106], Endiris et al. [107], and Bitana et al. [108], and also proposed livelihood diversification as a remedy to the severe food insecurity that disturbs rural households in Ethiopia and other parts of the world.

The results indicate that households engaging in a combination of farming and nonfarming livelihood diversification strategies experience improved food security compared with nondiversifier. This finding is statistically significant, demonstrating a clear link between these diversification strategies and improved food security. However, the average treatment effect estimation shows that combining farming with off-farming and nonfarming livelihood diversification strategies does not have a significant impact on the nutritional security status of farm households.

As noted by Duguma et al. [46], Sisay [88], Romeo et al. [109], Kandagor and Nyandoro [110], and Nkonde et al. [111], implementing farming and off-farming livelihood diversification strategies can improve nutrition security for farm households, as suggested by average treatment effect estimation. Furthermore, households that combine farming with nonfarming strategies also experience a notable improvement in nutrition security compared with those who do not diversify. Moreover, integrating farming with both off-farming and nonfarming strategies leads to a substantial enhancement in nutrition security for farm households compared with those who do not diversify.

The study shows significant transitional heterogeneity effects (TH) for nondiversifiers (HE0) and diversifiers (HE1), indicating differences between the 2 groups. Diversified households have higher food security (negative HE0 values) compared with nondiversified households. Transitional heterogeneity positively impacts livelihood diversification, with farm households engaging in off-farm and nonfarm activities showing a significant advantage over those that do not diversify. This is consistent with the earlier research conducted by Beyene et al. [112].

### Limitations and strengths

This study is limited in its geographical, content, and methodological scope in Ethiopia as it only focused on 2 districts. Therefore, the results and conclusions may not be generalizable to the entire country. In terms of limitations in data analysis methods, the MESR model frequently assumes static treatment effects, which may not adequately capture dynamic changes in treatment impact over time, particularly in longitudinal studies. Results from MESR are sensitive to the instrumental variables [113]. This study relies on cross-sectional data because there are no longitudinal data available from the study units. Future research should explore the use of longitudinal studies. Despite the aforementioned scope, and methodological limitations, the study did critically analyze the determinants of rural livelihood diversification strategies of farming households, and the impact of rural livelihood diversification strategies on food and nutrition security of farming households. The findings will underpin sustainable livelihood diversification strategies and food and nutrition security enhancement interventions by different stakeholders in the study area, and Ethiopia in the years to come. In other words, the findings will assist stakeholders of rural development sector in the study area and in similar agro ecologies in other parts of Ethiopia to address the sustainable development goals such as eradication of hunger, and sustainable management of local natural resources. It is hoped that more comprehensive and complementary studies may emerge with the replication of the innovative methods and new insights to address the pressing agricultural and development issues of the farming households in the study area, and in various parts of Ethiopia.

### Conclusion and policy recommendations

Food and nutrition security is a major issue affecting millions of people through the world, particularly in developing countries. In Ethiopia, like many other African countries, there is a critical need to enhance household food and nutrition security. The country’s food and nutrition insecurity is primarily attributed to its heavy dependence on limited and unproductive rain-fed agriculture. Farming is no longer the sole means of sustenance and economic growth for many rural households. Given their vulnerability to various shocks, it is essential to explore non/off-farm livelihood options. Therefore, the implementation of non/off-farm livelihood diversification strategies is vital for improving food and nutrition security among households in West Shoa, Ethiopia. Consequently, understanding the factors influencing livelihood diversification strategies and their impact on food and nutrition security in rural households in the region is crucial. This study aimed to investigate the determinants and effects of livelihood diversification strategies on food and nutrition security in the West Shoa Zone of Oromia, Ethiopia. The research utilized a combination of primary and secondary data sources. Primary data were collected from 385 sample households through semistructured questionnaire, whereas secondary data were gathered from various sources to complement the primary data.

Multinomial logit and multinomial endogenous switching models were used to analyze the factors affecting livelihood diversification and its impact on food and nutrition security. The findings from the multinomial logit model indicated that factors such as gender, age, family sizes, education level, livestock holding, social norms, land ownership, working capital, livelihood training, climate change information, access to extension services, policy, access to credit, farming experience, and proximity to weather roads play a significant role in determining the likelihood of adopting various levels of livelihood diversification strategies. The impact analysis revealed that households that combined farming with off-farm livelihood diversification strategies experienced a 75% enhancement in food security and a 42% improvement in nutrition security. Similarly, households that engaged in farming alongside nonfarming livelihood diversification strategies saw a 71% increase in food security and a 22% improvement in nutrition security compared with households that did not diversify their livelihoods. Integrating farming with both off-farm and nonfarming livelihood diversification strategies resulted in a 15% enhancement in nutrition security, although it did not significantly impact food security compared with households that did not diversify their livelihoods.

Our analysis of food and nutrition security measures based on the decision to participate in various diversification strategies reveals that the combined effect of using a mixed strategy or a combination of strategies is generally greater than the individual effects in most cases. This suggests that there is a potential synergy between diversification strategies, with combining diversification strategies leading to improved outcomes for farm households’ food and nutrition security. Therefore, to boost the food and nutrition security of smallholder farmers, the study recommends focusing on off-farm and nonfarm livelihood diversification in conjunction with farming activities rather than pursuing all diversification options simultaneously.

It is essential for policymakers, development practitioners, and researchers to comprehend these determinants to effectively support and promote sustainable livelihood diversification strategies that can improve the food and nutrition security of rural households. By taking these determinants into account, interventions and policies can be tailored to better meet the needs and capacities of small farm households and rural communities. Therefore, to enhance the food and nutrition security of smallholder farmers, it is crucial to focus on key factors that influence diversification decisions. This study suggests that improving smallholder farmers’ experience with livelihood diversification strategies should be a priority. This approach can help reduce the vulnerability of smallholder farmers to external shocks and enhance their overall resilience. Additionally, promoting access to markets, financial services, and agricultural extension services can further support smallholder farmers in improving their food and nutrition security.

The research emphasizes that social norms present major obstacles for smallholder farm households looking to expand their livelihoods into off/nonfarm activities. These norms deter smallholders from engaging in off/nonfarm activities. To support sustainable livelihood diversification and strengthen community resilience, it is crucial to tackle these detrimental norms through community involvement, education, and inclusive policies. Recognizing these factors is key to promoting sustainable agricultural practices and bolstering the resilience of smallholder communities. Initiatives to encourage diversification should take into account local cultural norms, current social frameworks, and opportunities for community involvement and empowerment.

Overall, livelihood diversification can have a significant impact on food and nutrition security. When household engaged in varieties of income-generating activities, they are less reliant on a single source of income, which can help buffer against economics shocks and improve overall resilience. This in turn can lead to improve access to food and better nutrition outcomes for smallholder farm households. Government and policymakers should recognize and support off- and nonfarm livelihood diversification strategies as part of the study area’s job creation objectives instead of increasing rural income and reducing rural poverty, which strongly relies on the development of off- or nonfarm activities. These results suggest that policymakers should support and promote livelihood diversification strategies to exploit the productivity effects of livelihood diversification strategies.

## Author contributions

The authors’ responsibilities were as follows – FH: prepared the proposal, collected and analyzed the data, interpreted the results, and wrote the paper; JHM, CSA, TTM: made substantial revisions to the content, had primary responsibility for final content; and all authors: read and approved the final manuscript.

## Data availability

Data will be made available on request.

## Conflict of interest

FH declares no known competing financial interests or personal relationships that could have appeared to influence the work reported in this paper. The other authors declare that they have no known competing financial interests or personal relationships that could have appeared to influence the work reported in this paper.
